# Complete Genome Sequences of Four Salmonella enterica Strains Associated with Pistachios Assembled Using a Combination of Short- and Long-Read Sequencing

**DOI:** 10.1128/MRA.00975-19

**Published:** 2019-09-19

**Authors:** Julie Haendiges, Narjol Gonzalez-Escalona, Jesse D. Miller, Maria Hoffmann

**Affiliations:** a Applied Research Center, NSF International, Ann Arbor, Michigan, USA; b Center for Food Safety and Applied Nutrition, Office of Regulatory Science, Division of Microbiology, U.S. Food and Drug Administration, College Park, Maryland, USA; Portland State University

## Abstract

Here, we report the genomes of two Salmonella enterica subsp. enterica serovar Montevideo strains (CFSAN005645 and FCC0123) and two Salmonella enterica subsp. *enterica* serovar Senftenberg strains (FSW0104 and CFSAN087304) isolated from pistachios. The genomes were closed using a hybrid assembly method using short- and long-read sequencing technology.

## ANNOUNCEMENT

Salmonella enterica is a Gram-negative bacterium that is responsible for an average of 1.2 million illnesses (1). Foods with low water activity, such as pistachios and other varieties of nuts, are becoming a vehicle for increasing numbers of these infections (2).

These four strains were selected for sequencing because they showed relatedness to each other based on results from the NCBI pathogen detection site (https://www.ncbi.nlm.nih.gov/pathogens/) and they were available in the lab. The genomes of these four strains from pistachios were sequenced to create reference strains in the event of future outbreaks. The Salmonella enterica subsp. enterica serovar Senftenberg strains, according to the NCBI pathogen detection site, belong to NCBI single nucleotide polymorphism (SNP) cluster PDS000031739. The Salmonella enterica subsp. enterica serovar Montevideo strains belong to NCBI SNP cluster PDS000032600. These clusters contain numerous isolates from pistachio and environmental samples over a 2- to 5-year timespan. These are all draft genomes with many contigs; therefore, to generate better reference genome sequences for these strains, their genomes were closed using a hybrid assembly method utilizing both short- and long-read sequence technology.

The strains were obtained by the U.S. Food and Drug Administration as part of a federal public health investigation. The strains were cultured in Trypticase soy broth (Becton, Dickinson, Franklin Lakes, NJ) overnight at 37°C. The genomic DNA was isolated using the Maxwell RSC cultured cells DNA kit (Promega, Madison, WI) following the manufacturer’s protocols, with the addition of RNase A treatment. The long-read sequencing was performed using a GridION device (Oxford Nanopore Technologies, Oxford, UK). The sequencing libraries were prepared using the Rapid Barcoding Sequencing kit (SQK-RBK004) following the manufacturer’s protocols. The prepared libraries were sequenced on a FLO-MIN106 (R9.4.1) flowcell for 48 hours. The long reads were base called and demultiplexed using Guppy v3.2.2. All reads below 5,000 base pairs in length were discarded. The short-read sequence data for each strain were previously generated by FDA GenomeTrakr-participating laboratories using a MiSeq instrument with 2 × 250-bp paired-end chemistry (Illumina, San Diego, CA). The short-read sequencing data were downloaded from the NCBI Sequence Read Archive. Each sample was individually analyzed by a previously published method (3) as follows: the long-read sequencing data were initially *de novo* assembled using Canu v1.7 (4) with default settings. A second assembly was produced using the SPAdes assembler (Galaxy v3.11.1) (5) with the hybrid option of using both short- and long-read sequencing outputs with default settings. Overlapping regions were identified at the end of the assemblies using Gepard and trimmed from the assembly (6). After comparison of the two assemblies for synteny using Mauve v20150226 (7), a final assembly was generated. A single circular, closed contig was generated for each isolate.

The four final assemblies were annotated using the NCBI Prokaryotic Genome Annotation Pipeline and deposited at DDBJ/EMBL/GenBank. The sequencing statistics can be found in Table 1. *In silico* multilocus sequence typing (MLST) analyses (8) showed two sequence types (STs); the two *S*. Montevideo strains belong to ST138, and the two *S*. Senftenberg strains belong to ST185. The *S*. Montevideo strains belong to clade I of the four clades previously described for *S.* Montevideo and to eBurstGroup 39 (eBG 39) (9). The other three clades belong to eBG 40, which contains the majority of the *S*. Montevideo strains. The *S*. Senftenberg strains belong to eBG 30, whereas the majority of *S.* Senftenberg strains are in eBG 55 (8).

**TABLE 1:** Metadata of the strains sequenced in this study

Isolate (BioSample accession no.)	No. of long reads (>4,999 bp)	Collection yr	NCBI accession no. (genome length [bp])	G+C content (%)	Hybrid assembly coverage (×)	SRA accession no.	
						Short read	Long read
CFSAN005645 (SAMN02265272)	167,338	2009	CP040380 (4,615,193)	52.3	103	SRR958039	SRR9052981
FCC0123 (SAMN02678532)	194,831	2009	CP040379 (4,619,529)	52.3	73	SRR5384655	SRR9052982
FSW0104 (SAMN02678831)	33,310	2013	CP037894 (4,820,913)	52.2	77	SRR1257282	SRR8707510
CFSAN087304 (SAMN10498876)	43,679	2018	CP037892 (4,819,673)	52.2	25	SRR8261878	SRR8707509

### Data availability.

The genome sequences of the four S. enterica strains are listed in Table 1.
